# Supplementary material for: PIP2-dependent coupling of voltage sensor and pore domains in Kv7.2 channel
Source: Commun Biol. 2021 Oct 14;4:1189. doi: 10.1038/s42003-021-02729-3 (PMC8517023; doi:10.1038/s42003-021-02729-3)
Supplement: Supplementary file 1 — Supplementary Information [file 42003_2021_2729_MOESM1_ESM.pdf]

SUPPLEMENTARY INFORMATION

PIP$_2$-dependent coupling of voltage sensor and pore domains in K$_V$7.2 channel

Shashank Pant[1,2,3,‡], Jiaren Zhang[4,‡], Eung Chang Kim[4,‡], Kin Lam[1,2,5], Hee Jung Chung[4,6,*], Emad Tajkhorshid[1,2,3,6,*]

[1]Theoretical and Computational Biophysics Group, NIH Center for Macromolecular Modeling and Bioinformatics, Beckman Institute for Advanced Science and Technology, University of Illinois at Urbana-Champaign, Urbana, IL 61801, USA, [2]Department of Biochemistry, University of Illinois at Urbana-Champaign, Urbana, IL 61801, USA, [3]Center for Biophysics and Quantitative Biology, University of Illinois at Urbana-Champaign, Urbana, IL 61801, USA, [4]Department of Molecular and Integrative Physiology, University of Illinois at Urbana-Champaign, Urbana, IL 61801, USA, [5]Department of Physics, University of Illinois at Urbana-Champaign, Urbana, IL 61801, USA, [6]Neuroscience Program, University of Illinois at Urbana-Champaign, Urbana, IL 61801, USA.

[‡]These authors contributed equally.

[*]These authors jointly supervised this work

CORRESPONDING AUTHORS:

Emad Tajkhorshid
Beckman Institute for Advanced Science and Technology,
University of Illinois at Urbana-Champaign,
405 N Mathews Avenue, 3147 Beckman Institute,
Urbana, IL, 61801, USA.
Email: emad@illinois.edu
Tel: (217) 244-6914

Hee Jung Chung
Department of Molecular and Integrative Physiology,
University of Illinois at Urbana-Champaign,
407 South Goodwin Avenue, 524 Burrill Hall,
Urbana, IL 61801, USA.
Email: chunghj@life.illinois.edu
Tel: (217) 244-6839

SUPPLEMENTARY FIGURES

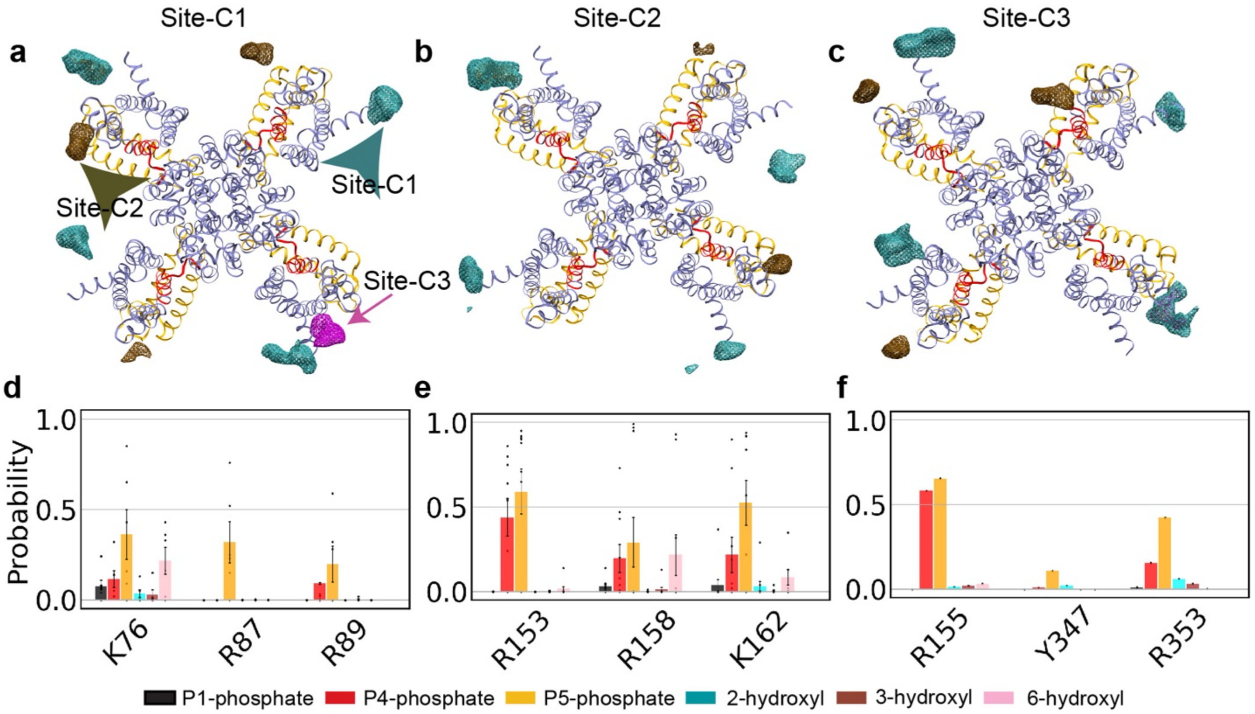

Figure S1. PIP$_2$ coordination in the 3 binding sites captured during the simulation of the recent cryo-EM structure of the closed K$_v$7.2 channel. Three independent simulations were performed on the recently published cryo-EM structure of a tetrameric human K$_v$7.2 channel in a closed state (PDB ID: 7CR3)[1] after removing calmodulin. A 4 Å heavy-atom distance cutoff was used to define a contact between the protein residues and the phosphate groups of PIP$_2$, whereas a 3.5 Å cutoff was used to define a contact between the residues and the hydroxyl groups of PIP$_2$. Three PIP$_2$ binding sites were identified: Site-C1, Site-C2, and Site-C3 (where C stands for a "closed" state). **(a-c)** PIP$_2$ headgroup occupancy maps extracted from the individual simulation trajectories is shown as a wireframe overlaid on a snapshot taken from Run1 **(a)**, Run2 **(b)**, and Run3 **(c)**. Consistent with the simulation results on the modeled tetrameric closed K$_v$7.2 channel (Fig. 1c), PIP$_2$ binds to Site-C1 (the N-terminal tail), Site-C2 (the S2-S3 linker), and Site-C3 (at the interface formed by Helix-A, and the S2-S3 and AB linkers) in all simulation runs. **(d-f)** The contact probability for each chemical moiety of PIP$_2$ headgroup with the key residues in the binding Site-C1 **(a)**, Site-C2 **(b)**, and Site-C3 **(c)**. The chemical moieties of PIP$_2$ headgroup include P1-phosphate (black), P4-phosphate (red), P5-phosphate (orange), 2-hydroxyl (cyan), 3-hydroxyl (brown), and 6-hydroxyl (pink) group of the inositol ring. Analysis of the contact probabilities was performed over the last 200 ns of simulation trajectories. The number of PIP$_2$ interacting events: Site-C1 (n=6), Site-C2 (n=7), and Site-C3 (n=1). Data represent mean ± SEM for 3 independent simulation trajectories.

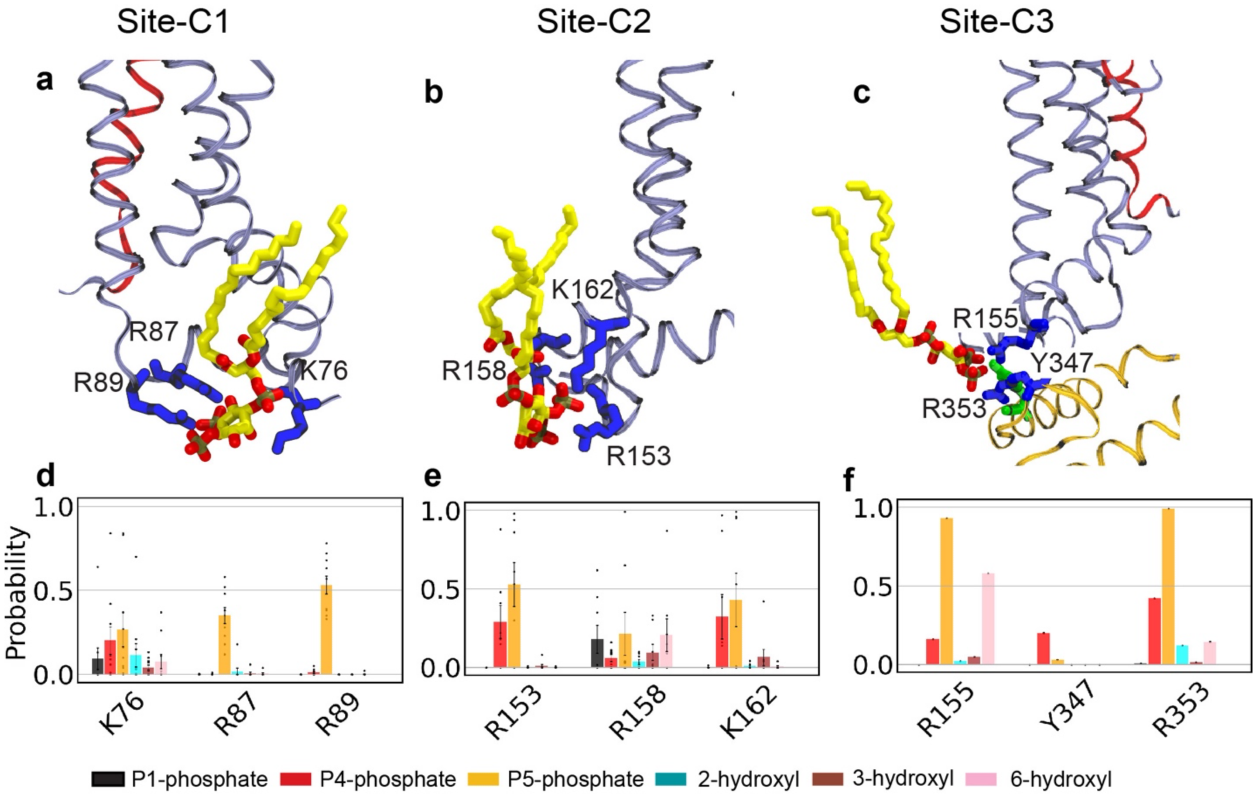

Figure S2. PIP_2 coordination in the 3 binding sites captured in the closed $K_v7.2$ channel. Three independent simulations were performed in the modeled structure of a closed $K_v7.2$ channel in the absence of calmodulin. A tetrameric human $K_v7.2$ channel in a closed state was modeled from the cryo-EM structure of human $K_v7.1$ channel with a closed pore (PDB ID: 5VMS). A 4 Å heavy-atom distance cutoff was used to define a contact between a residue and the phosphate groups of PIP_2, whereas a 3.5 Å cutoff was used to define a contact between a residue and the hydroxyl groups of PIP_2. **(a-c)** PIP_2 coordination is shown for Site-C1 **(a)**, Site-C2 **(b)**, and Site-C3 **(c).** $K_v7.2$ is shown in a ribbon representation with S4 in red, helices A and B in brown, and the rest of the protein in ice blue. A PIP_2 lipid (carbon atoms in yellow, oxygen in red, and phosphorus in tan) and the residues of the binding pocket at each site at the end of the simulation are shown in sticks (basic residues in blue, polar in green, acidic in red, and hydrophobic in white). **(d-f)** The contact probability for each chemical moiety of PIP_2 headgroup with the key residue in the binding Site-C1 **(d)**, Site-C2 **(e)**, and Site-C3 **(f)**. The chemical moieties of PIP_2 headgroup include P1-phosphate (black), P4-phosphate (red), P5-phosphate (orange), 2-hydroxyl (cyan), 3-hydroxyl (brown), and 6-hydroxyl (pink) group of the inositol ring. Analysis of the contact probabilities was performed over the last 200 ns of simulation trajectories. The number of PIP_2 interacting events: Site-C1 (n=10), Site-C2 (n=7), and Site-C3 (n=1). Data represents mean ± SEM for 3 independent trajectories.

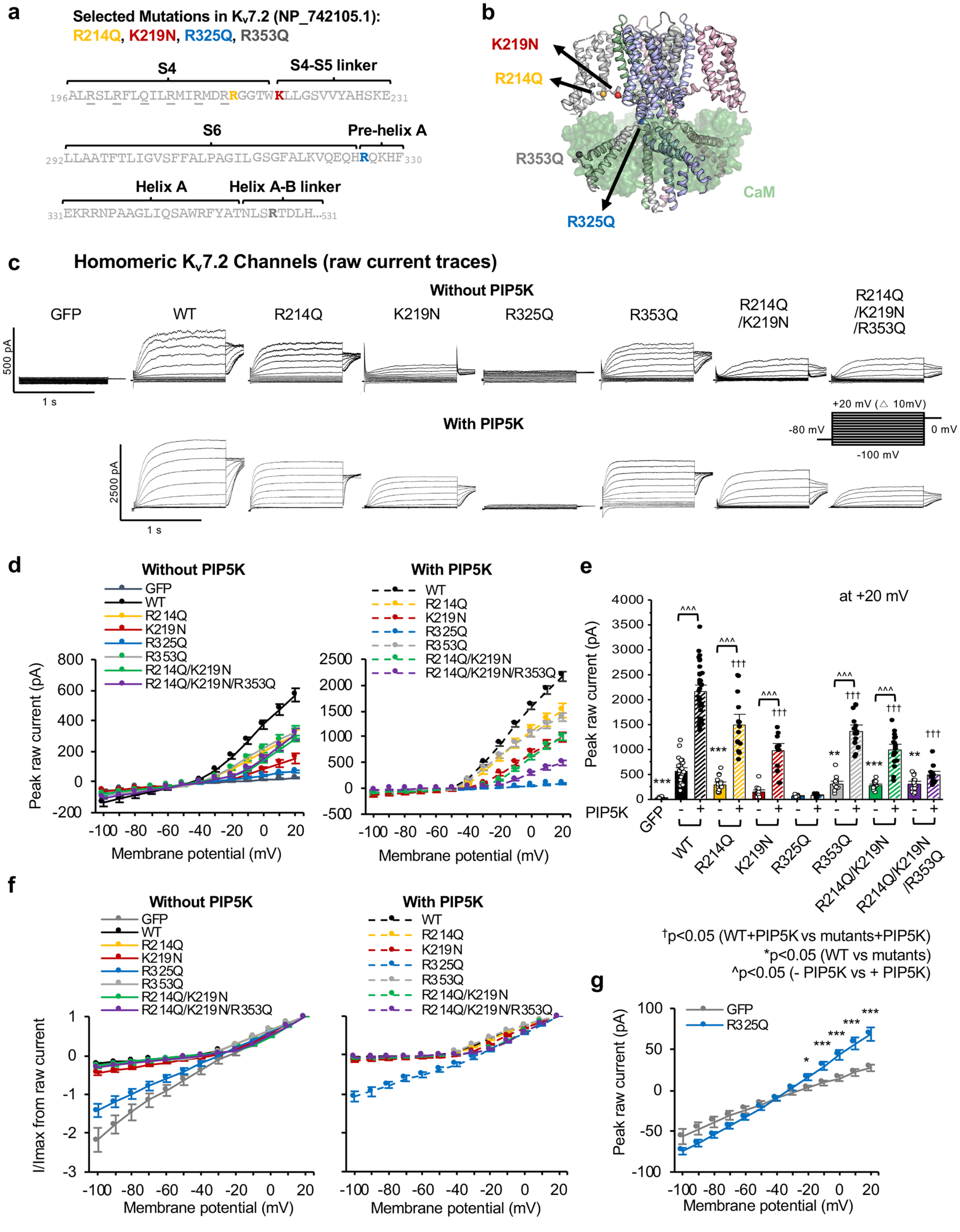

a
Selected Mutations in Kv7.2 (NP_742105.1):
R214Q, K219N, R325Q, R353Q
S4
S4-S5 linker
196ALRSLRFLQILRMIRMDRRGGTWKLLGSVVYAHSKE231
S6
Pre-helix A
292LLAATFTLIGVSFFALPAGILGSGFALKVQEQHRQKHF330
Helix A
Helix A-B linker
331EKRRNPAAGLIQSAWRFYATNLSRTDLH...531
b
K219N
R214Q
R353Q
CaM
R325Q
c
Homomeric Kv7.2 Channels (raw current traces)
Without PIP5K
GFP
WT
R214Q
K219N
R325Q
R353Q
R214Q /K219N
R214Q /K219N /R353Q
500 pA
1 s
With PIP5K
+20 mV (△ 10mV)
-80 mV
0 mV
-100 mV
2500 pA
1 s
d
Without PIP5K
With PIP5K
GFP
WT
R214Q
K219N
R325Q
R353Q
R214Q/K219N
R214Q/K219N/R353Q
Peak raw current (pA)
Membrane potential (mV)
e
at +20 mV
Peak raw current (pA)
PIP5K
GFP
WT
R214Q
K219N
R325Q
R353Q
R214Q/K219N
R214Q/K219N /R353Q
†p<0.05 (WT+PIP5K vs mutants+PIP5K)
*p<0.05 (WT vs mutants)
^p<0.05 (- PIP5K vs + PIP5K)
f
Without PIP5K
With PIP5K
I/Imax from raw current
Membrane potential (mV)
g
GFP
R325Q
Peak raw current (pA)
Membrane potential (mV)

Figure S3. Charge-neutralizing mutations of potential PIP_2 binding residues affect voltage- and PIP_2-dependent activation of homomeric K_v7.2 channels. (a) Locations of selected mutations (R214Q, K219N, R325Q, and R353Q) are highlighted in the amino acid sequence of K_v7.2 (NP_742105.1). The critical residues in the S4 are underlined. **(b)** The Cα atoms of the mutation sites are highlighted in one subunit of the modeled tetrameric human K_v7.2 structure (ribbons) in complex with four CaM subunits (transparent green surfaces). **(c-f)** Whole cell patch clamp recordings of K^+ currents through WT or mutant K_v7.2 channels in GFP-positive CHO hm1 cells in the presence or absence of PIP5K cotransfection. CHO hm1 cells were co-transfected with 0.2 μg GFP, 0.45 μg K_v7.2/*KCNQ2* WT or mutant, and 0.45 μg PIP5K DNA. Cells were held at −80 mV and K^+ currents were evoked by depolarizing voltage steps for 1.5 s from −100 mV to +20 mV in 10-mV increments, followed by a step to 0 mV for 300 ms. **(c)** Representative raw current traces. **(d-e)** Average peak current is quantified from raw currents at all voltage steps **(d)** and +20 mV **(e)**. **(f)** Normalized currents (I/I_{max}) are quantified using raw currents at all voltage steps. **(g)** Average peak raw currents in GFP-transfected cells versus those in cell transfected with GFP and K_v7.2-R325G at all voltage steps. The source data for Fig. S3d, f, and g are available in DOI: 10.6084/m9.figshare.15181038. Data represent mean ± SEM. One-way ANOVA with post-hoc Fisher's multiple comparison test was used. Leak subtracted current traces and data analysis are shown in Fig. 4 of the main text.

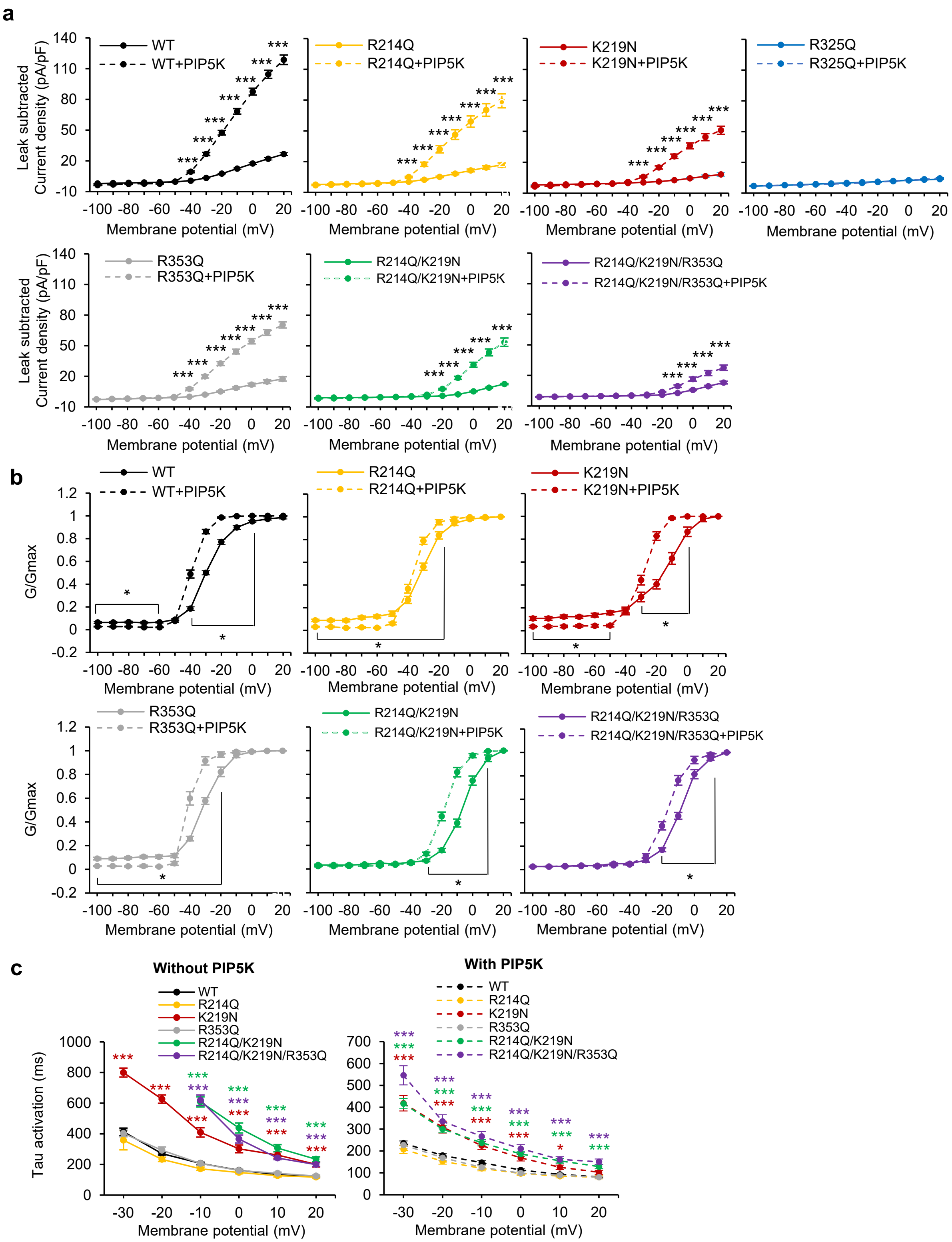
a
WT
WT+PIP5K
R214Q
R214Q+PIP5K
K219N
K219N+PIP5K
R325Q
R325Q+PIP5K
R353Q
R353Q+PIP5K
R214Q/K219N
R214Q/K219N+PIP5K
R214Q/K219N/R353Q
R214Q/K219N/R353Q+PIP5K
Leak subtracted Current density (pA/pF)
Membrane potential (mV)
b
G/Gmax
Membrane potential (mV)
c
Without PIP5K
With PIP5K
Tau activation (ms)
Membrane potential (mV)

Figure S4. Charge-neutralizing mutations of potential PIP_2 binding residues affect PIP5K-dependent modulation of current potentiation and voltage dependence of K_v7.2 channels. Whole cell patch clamp recordings of K^+ currents through WT or mutant K_v7.2 channels in GFP-positive CHO hm1 cells with or without PIP5K cotransfection. **(a-c)** The data from Figure 4 are replotted to show the effects of PIP5K cotransfection on the current expression of K_v7.2 WT or each mutant channel. **(a)** Average peak current densities, **(b)** normalized conductance (G/G_{max}), **(c)** activation time constant (τ) of K_v7.2 WT or mutant channels with or without PIP5K coexpression at all voltage steps. The source data for Fig. S4a, b, and c are available in DOI: 10.6084/m9.figshare.15181038. Data represent mean ± SEM. Student's t-test was used for panels a-b and one-way ANOVA with post-hoc Fisher's multiple comparison test is used in panel c. *$p<0.05$, **$p<0.01$, ***$p<0.005$.

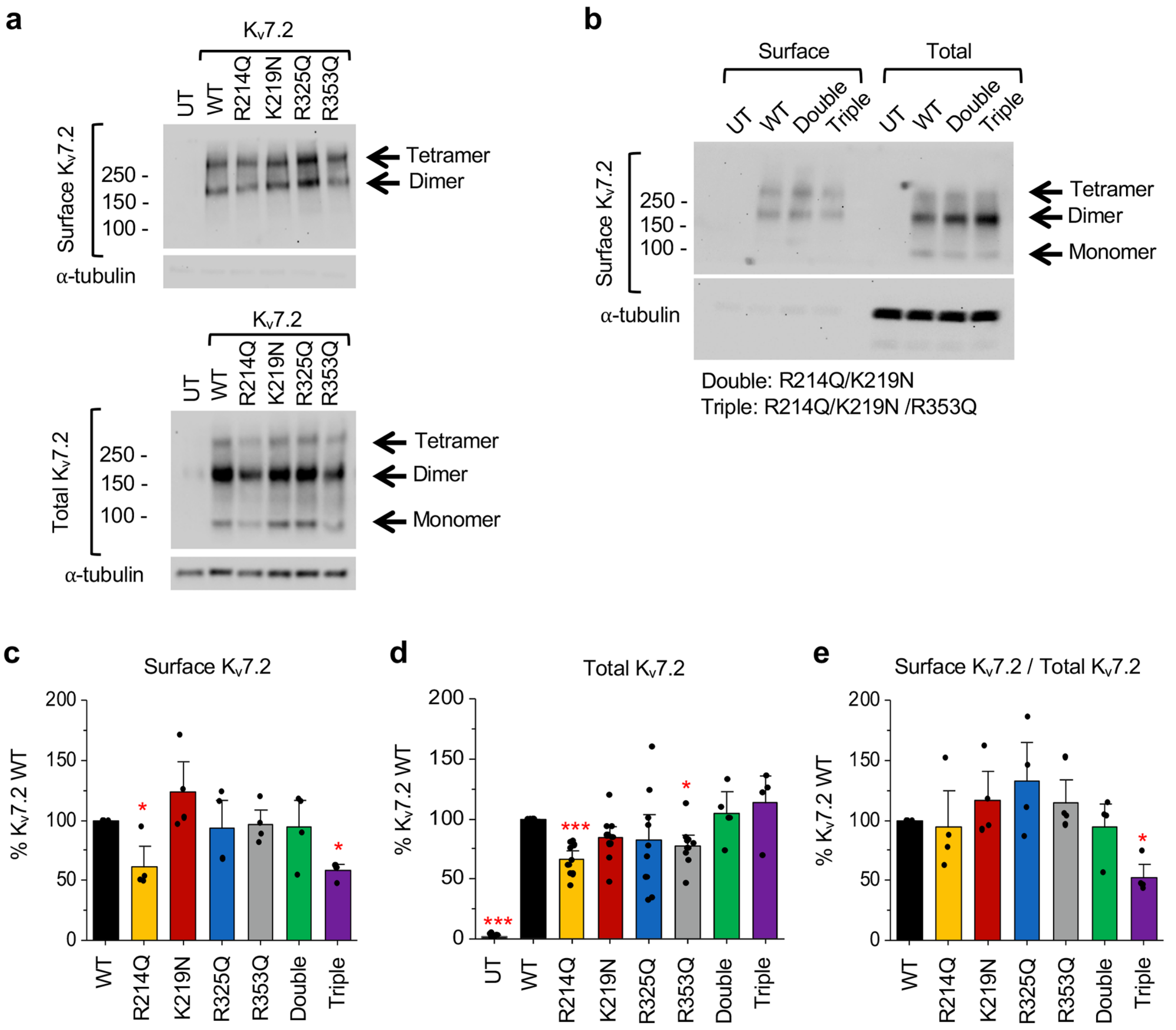

Figure S5. Charge-neutralizing mutations of potential PIP$_2$ binding residues are expressed on the cell surface of CHO hm1 cells. Surface biotinylation was performed on live CHO hm1 cells with Sulfo-NHS-SS-Biotin. Surface biotinylated proteins were isolated by NeutrAvidin agarose beads, released from the beads by a 30-min incubation in the Sample Buffer at 75 °C, and analyzed by immunoblot analysis with antibodies for $K_v7.2$ and the loading controls (GAPDH, α-tubulin, or β-tubulin). **(a-b)** Representative immunoblot images of surface and total $K_v7.2$ WT and mutant proteins as well as β-tubulin. Untransfected cells (UT) served as a negative control. In total lysate (bottom blot), WT and mutant $K_v7.2$ proteins were expressed as monomers, dimers, and tetramers as we have previously reported [14]. In surface biotinylated fractions (top blots), $K_v7.2$ proteins were detected only as dimers and tetramers. The lack of monomeric bands is due to the extended exposure to the denaturing Sample Buffer, which facilitates oligomerization of $K_v7.2$ proteins. **(c)** Quantification of surface $K_v7.2$, which was normalized to total α-tubulin and presented as % $K_v7.2$ WT (n=4 dishes for each construct). **(d)** Total $K_v7.2$ level was normalized to the loading controls (GAPDH, α-tubulin, or β-tubulin). The number of dishes containing transfected cells: WT (n=9), single mutations (n=9), the double-mutant R214Q/K219N (n=4), and the triple-mutant R214Q/K219N/R353Q (n=4). **(e)** Quantification of the "surface $K_v7.2$ / total $K_v7.2$" ratio, which was normalized to the WT ratio (WT as 100%, n=4 dishes for each construct). Data represent the mean ± SEM. One-way ANOVA with post-hoc Fisher's test was conducted. *$p<0.05$, **$p<0.01$, ***$p<0.005$.

SUPPLEMENTARY TABLES

Table S1. Passive properties of CHO hm1 cells transfected with GFP, $K_v7.2$, and PIP5K.

	Without PIP5K			*With PIP5K*		
Transfection	*n*	V_m (mV)	C_m (pF)	*n*	V_m (mV)	C_m (pF)
GFP	12	-14.4 ± 1.2*	17.3 ± 0.3	NA	NA	NA
GFP + WT	31	-40. ± 0.5	17.7 ± 0.2	33	-50.1 ± 0.4^	17.8 ± 0.2
GFP + R214Q	16	-39.5 ± 1.0	17.7 ± 0.2	15	-47.2 ±0.7^	18.8 ± 0.2
GFP + K219N	12	-37.0 ± 0.7*	18.0 ± 0.3	12	-41.6 ± 1.0^†	18.9 ± 0.5
GFP + R325Q	11	-39.1 ± 0.4	17.8 ± 0.3	11	-40.6 ± 1.2†	17.7 ± 0.4
GFP + R353Q	13	-40.4 ± 1.0	17.7 ± 0.1	16	-49.3 ± 0.8^	19.4 ± 0.4
GFP+ R214Q/K219N	15	-31.1 ± 0.7*	17.6 ± 0.2	19	-39.9 ± 0.7^†	17.4 ± 0.1
GFP+ R214Q/K219N/R353Q	14	-25.7 ± 5.0*	17.5 ± 0.1	14	-36.7 ± 1.2^†	17.5 ± 0.1

n, number; NA, not applicable; V_m, resting membrane potential; C_m, whole cell membrane capacitance; Mean ± SEM (GFP+ selected variant: *p<0.05 for $K_v7.2$ WT vs. mutant; †p<0.05 for $K_v7.2$ WT + PIP5K vs. mutants + PIP5K; ^p<0.05 for the difference between -PIP5K and +PIP5K within the same transfection. The source data for Table S1 is available in DOI: 10.6084/m9.figshare.15181038.

SUPPLEMENTARY REFERENCE

1 Li, X. *et al.* Molecular basis for ligand activation of the human KCNQ2 channel. *Cell Res* **31**, 52-61, doi:10.1038/s41422-020-00410-8 (2021).
