# Supplementary material for: PIP2-dependent coupling of voltage sensor and pore domains in Kv7.2 channel
Source: Commun Biol. 2021 Oct 14;4:1189. doi: 10.1038/s42003-021-02729-3 (PMC8517023; doi:10.1038/s42003-021-02729-3)
Supplement: Supplementary file 2 — Description of Additional Supplementary Files [file 42003_2021_2729_MOESM2_ESM.pdf]

Description of Additional Supplementary Files

File name: Supplementary Movie 1-3.
Description: PIP2 binding to Kv7.2 in the open state. Starting from the unbound form, three independent 500-ns MD simulations captured binding of PIP2 to R353, which acts as an initial anchor point, after which the lipid forms contacts with the other residues in Site-O1 (the interface of the S2-S3 and AB linkers).

File name: Supplementary Movie 4.
Description: PIP2-induced conformational changes in the intracellular helices A and B of Kv7.2 channels. Large-scale conformational changes in the cytoplasmic helices A and B, which are induced by the presence of anionic lipids in the membrane, in turn facilitate the movement of PIP2 along Helix-B, ultimately leading to its binding to Site-O4 (the interface of S4, the S4-S5 linker, S6, and pre-Helix-A).
