# Supplementary material for: PIP2-dependent coupling of voltage sensor and pore domains in Kv7.2 channel
Source: Commun Biol. 2021 Oct 14;4:1189. doi: 10.1038/s42003-021-02729-3 (PMC8517023; doi:10.1038/s42003-021-02729-3)
Supplement: Supplementary file 7 — Reporting Summary [file 42003_2021_2729_MOESM7_ESM.pdf]

nature research

Corresponding author(s): Hee Jung Chung and Emad Tajkhorshid

Last updated by author(s): Sep 20, 2021

Reporting Summary

Nature Research wishes to improve the reproducibility of the work that we publish. This form provides structure for consistency and transparency in reporting. For further information on Nature Research policies, see our Editorial Policies and the Editorial Policy Checklist.

Statistics

For all statistical analyses, confirm that the following items are present in the figure legend, table legend, main text, or Methods section.

n/a	Confirmed	
[ ]	[x]	The exact sample size (*n*) for each experimental group/condition, given as a discrete number and unit of measurement
[ ]	[x]	A statement on whether measurements were taken from distinct samples or whether the same sample was measured repeatedly
[ ]	[x]	The statistical test(s) used AND whether they are one- or two-sided<br>*Only common tests should be described solely by name; describe more complex techniques in the Methods section.*
[x]	[ ]	A description of all covariates tested
[x]	[ ]	A description of any assumptions or corrections, such as tests of normality and adjustment for multiple comparisons
[ ]	[x]	A full description of the statistical parameters including central tendency (e.g. means) or other basic estimates (e.g. regression coefficient) AND variation (e.g. standard deviation) or associated estimates of uncertainty (e.g. confidence intervals)
[x]	[ ]	For null hypothesis testing, the test statistic (e.g. *F*, *t*, *r*) with confidence intervals, effect sizes, degrees of freedom and *P* value noted<br>*Give P values as exact values whenever suitable.*
[x]	[ ]	For Bayesian analysis, information on the choice of priors and Markov chain Monte Carlo settings
[x]	[ ]	For hierarchical and complex designs, identification of the appropriate level for tests and full reporting of outcomes
[x]	[ ]	Estimates of effect sizes (e.g. Cohen's *d*, Pearson's *r*), indicating how they were calculated

Our web collection on statistics for biologists contains articles on many of the points above.

Software and code

Policy information about availability of computer code

Data collection	The iBright CL1000 imaging system built-in software (Invitrogen).<br>pClamp 10.6 (Molecular Devices)<br>Clampfit 10.6 (Molecular Devices)<br>Simulation trajectories were collected using NAMD, which is publicly available.
Data analysis	Image J (National Institute of Health)<br>Origin9.1 (OriginLab, Inc)<br>Microsoft Excel<br>Visualization and analysis of the simulation results were performed using VMD and Python, both publicly available.

For manuscripts utilizing custom algorithms or software that are central to the research but not yet described in published literature, software must be made available to editors and reviewers. We strongly encourage code deposition in a community repository (e.g. GitHub). See the Nature Research guidelines for submitting code & software for further information.

Data

Policy information about availability of data

All manuscripts must include a data availability statement. This statement should provide the following information, where applicable:

- Accession codes, unique identifiers, or web links for publicly available datasets
- A list of figures that have associated raw data
- A description of any restrictions on data availability

The data that support the findings of this study are available from the corresponding authors upon reasonable request.

Field-specific reporting

Please select the one below that is the best fit for your research. If you are not sure, read the appropriate sections before making your selection.

☒ Life sciences ☐ Behavioural & social sciences ☐ Ecological, evolutionary & environmental sciences

For a reference copy of the document with all sections, see nature.com/documents/nr-reporting-summary-flat.pdf

Life sciences study design

All studies must disclose on these points even when the disclosure is negative.

Sample size	The sample sizes (n) for the western blots (n = 4-10) and electrophysiology experiments (n = 11 - 20) in CHOhm1 cells were determined based on our previous publications (PMC7075958, PMC6415549).
Data exclusions	No data was excluded from the analysis.
Replication	Measures taken to verify the reproducibility of the western blots and electrophysiology findings were (i) to conduct at least 3 repeat experiments, and (ii) to increase sufficient number of sample sizes which were determined based on our previous publications.
Randomization	Randomization is not relevant to this study. For every experiment, we compared WT vs. mutant by introducing them to CHO hm1 cells that were cultured at the same density, using the same media, from the same maintenance plate.
Blinding	For surface biotinylation study, lysate samples from cells transfected with WT or mutant were loaded side-by-side and were subject to the exact same procedures in western blot (antibodies, incubation time, number of washing).

Reporting for specific materials, systems and methods

We require information from authors about some types of materials, experimental systems and methods used in many studies. Here, indicate whether each material, system or method listed is relevant to your study. If you are not sure if a list item applies to your research, read the appropriate section before selecting a response.

Materials & experimental systems

n/a	Involved in the study	
☐	☒	Antibodies
☐	☒	Eukaryotic cell lines
☒	☐	Palaeontology and archaeology
☒	☐	Animals and other organisms
☒	☐	Human research participants
☒	☐	Clinical data
☒	☐	Dual use research of concern

Methods

n/a	Involved in the study	
☒	☐	ChIP-seq
☒	☐	Flow cytometry
☒	☐	MRI-based neuroimaging

Antibodies

Antibodies used	Primary antibodies used include anti-GAPDH (Cell Signaling #2118, 1:1000 dilution), anti-a-tubulin (Cell Signaling #2144, 1:1000 dilution), anti-β-tubulin (Cell Signaling #2146, 1:1000), anti-Kv7.2 (Neuromab, N26A/23, 1:200 dilution). Secondary antibodies include donkey anti-rabbit and donkey anti-mouse HRP secondary antibodies (The Jackson Laboratory, 711-035-152; 715-035-150).
Validation	Anti-Kv7.2 antibody (Neuromab, N26A/23) has been validated by our lab using cell lysates from CHO cells transfected with pcDNA3-KCNQ2 plasmid.<br>All Cell Signaling antibodies (anti-a-tubulin, #2144; anti-β-tubulin, #2146; anti-GAPDH, #2118) have been validated by numerous labs. Validation data can be found on the Cell Signaling website.<br>anti-a-tubulin: https://www.cellsignal.com//products/primary-antibodies/a-tubulin-antibody/2144<br>anti-β-tubulin: https://www.cellsignal.com//products/primary-antibodies/b-tubulin-antibody/2146<br>anti-GAPDH: https://www.cellsignal.com/products/primary-antibodies/gapdh-14c10-rabbit-mab/2118

Eukaryotic cell lines

Policy information about cell lines

Cell line source(s)	CHO hm1 (Source: Naoto Hoshi, UC Irvine)
Authentication	This cell line is stably transfected with cDNA encoding the human M1 muscarinic acetylcholine receptor. It was produced and authenticated by Mullaney et al, 1993 (PMC1132139). This cell line has been widely used and authenticated for the

	muscarinic regulation of Kv7 channels by many different labs including Naoto Hoshi's group (PMC6782456; PMC3400014)
Mycoplasma contamination	Cell line was not tested for mycoplasma contamination
Commonly misidentified lines (See ICLAC register)	N/A
